# A multicenter, randomized, double-blind trial of a new porcine surfactant in premature infants with respiratory distress syndrome

**DOI:** 10.1590/S1679-45082014AO3095

**Published:** 2014

**Authors:** Celso Moura Rebello, Alexander Roberto Precioso, Renata Suman Mascaretti, Grupo Colaborativo do Estudo Brasileiro Multicêntrico de Surfactante

**Affiliations:** 1 Hospital Israelita Albert Einstein, São Paulo, SP, Brazil.; 2 Instituto da Criança, Hospital das Clínicas, Faculdade de Medicina, Universidade de São Paulo, São Paulo, SP, Brazil.; 3 Maternidade Pro Matre Paulista, São Paulo, SP, Brazil.

**Keywords:** Pulmonary surfactants/therapeutic use, Newborn, very low birth weight, Newborn, Preterm, Respiratory distress syndrome, newborn/drug therapy

## Abstract

**Objective:**

To compare the efficacy and safety of a new porcine-derived pulmonary surfactant developed by *Instituto Butantan* with those of animal-derived surfactants commercially available in Brazil, regarding neonatal mortality and the major complications of prematurity in preterm newborns with birth weight up to 1500g and diagnosed with respiratory distress syndrome.

**Methods:**

Neonates diagnosed with respiratory distress syndrome were randomized to receive either Butantan surfactant (Butantan group) or one of the following surfactants: Survanta^®^ or Curosurf^®^. Newborns receiving Survanta^®^ or Curosurf^®^ comprised the control group. The main outcome measures were mortality rates at 72 hours and at 28 days of life; the typical complications of prematurity as evaluated on the 28th day of life were defined as secundary outcomes.

**Results:**

No differences were observed between the Butantan (n=154) and control (n=173) groups in relation to birth weight, gestational age, sex, and prenatal use of corticosteroids, or in mortality rates both at 72 hours (14.19% *versus* 14.12%; p=0.98) and at 28 days (39.86% *versus* 33.33%; p=0.24) of life. Higher 1- and 5-minute Apgar scores were observed among control group newborns. No differences were observed as regards the secondary outcomes, except for greater need for supplemental oxygen and a higher incidence of interstitial pulmonary emphysema in the Butantan group.

**Conclusion:**

The mortality rates at 72 hours and 28 days of life and the incidence of major complications of prematurity were comparable to those found with the animal-derived surfactants commercially available in Brazil, showing the efficacy and safety of the new surfactant in the treatment of respiratory distress syndrome in newborns.

## INTRODUCTION

The neonatal respiratory distress syndrome (RDS) is primarily caused by a qualitative and quantitative surfactant deficiency at birth. Avery and Mead are responsible for the pioneering studies relating surfactant deficiency to this condition.^(1)^

The first experimental study demonstrating the benefitial effect of surfactant replacement was conducted in the early 1970’s by Enhornig and Robertson,^(2)^ by means of the instillation of a natural surfactant, obtained from adult rabbits lungs, into the trachea of preterm rabbits. Subsequent studies have been conducted both to prove the efficacy of surfactants and to improve their extraction and purification. Some of the pioneers in clinical studies were Fujiwara et al.^(3)^ who proved, in a non-controlled study, improved oxygenation after surfactante administration in ten preterm newborns.

In the early 1990’s, surfactants started to be routinely used in several neonatal intensive care centers. Since then, the “optimal surfactant” – characterized by proven efficacy, safety, good resistance to inactivation by plasma proteins, and low cost, has been sought for routine use. The following functions are attributed to surfactants: preventing alveolar collapse at the end of expiration; secondarily decreasing alveolar edema; and showing actions related to immunity.^(4)^

Exogenous surfactant therapy allowed a reduction in neonatal mortality rates among extremely preterm newborns, with an impact also on infant mortality rates in developed countries. A limitation for its large-scale use in preterm infants as well as in older children and adults is its high cost. Thus, aiming at attenuating this problem nationwide, *Instituto Butantan*– located in the city of São Paulo (State of São Paulo), developed a new method of extraction and purification of a natural porcine-derived surfactant, and obtained a new product with a reduced production cost in relation to imported products.^(5,6)^

This new process was based on maceration of suine lungs followed by organic extraction coupled to adsortion in diethylaminoethyl cellulose (DEAE-C). This technology has the advantage of reducing the costs of production not only for not using large industrial-scale refrigerated ultracentrifuges, but also for reusing the reagents used in the different stages of extraction. Its chemical composition is quite similar to that of other animal-derived surfactants, with its lipid content represented by phosphatidylcholine (76%), phosphatidylethanolamine (6 to 8%), phosphatidylglycerol (6%), phosphatidylinositol (6%), and sphingomyelin (4 to 6%), in addition to surfactant proteins B and C (5.6%).^(5)^ The *in vivo* efficacy of this product was proven using an experimental RDS model.^(7)^ In view of the theoretical risk of immunogenicity to the proteins present in the pulmonary surfactant,^(8,9)^ the immune response to exposure to a new product was studied, and it was demonstrated, in an animal model, that the surfactant produced by *Instituto Butantan* was not associated with the occurence of clinical manifestations that could preclude its use in preterm newborns with RDS.^(10)^

The new surfactant was also used for the treatment of meconium aspiration syndrome in an animal model.^(11,12)^ The results of these studies demonstrated that the animals treated with the surfactant produced by *Instituto Butantan* showed significant improvement of their pulmonary mechanics, pulmonary volumes, and histopathological patterns of alveolar aeration.^(11,12)^ Based on these studies, we hypothesized that the surfactant produced by *Instituto Butantan* is as efficient and safe as the surfactants commercially available in Brazil for the treatment of neonatal RDS.

## OBJECTIVE

To evaluate the efficacy and safety of a new porcine-derived pulmonary surfactant developed by *Instituto Butantan* for the treatment of neonatal respiratory distress syndrome, in comparison to those of animal-derived surfactants available in the country, particularly in relation to neonatal mortality and the incidence of pulmonary hemorrhage and pneumothorax.

## METHODS

### Study population

We conducted a multicenter, prospective, randomized study including 19 neonatal intensive care units distributed in the States of São Paulo (6 centers), Rio de Janeiro (3 centers), Minas Gerais (3 centers), Sergipe (2 centers), Rio Grande do Sul (2 centers), Pernambuco (1 center), Distrito Federal (1 center), and Maranhão (1 center), from April 2005 to August 2007. Pregnant women at a high risk for preterm delivery (more than 24 weeks gestation and birth weight estimated at between 501 and 1,500g) were invited to participate in the study. If the inclusion criteria were met and the written informed consent was given, the newborn was considered eligible for randomization.

The inclusion criteria were: preterm neoneonates born in the study centers; with birth weight between 501 and 1,500g; postnatal age ≤24 hours of life; undergoing mechanical ventilation; with an arterial oxygen pressure and inspired oxygen fraction ratio (PaO_2_/FiO_2_) ≤175; clinically and radiologically diagnosed with RDS; and written informed consent given by parents or guardians.

The exclusion criteria were: previous treatment with another type of surfactant; newborn with major congenital malformations or previously diagnosed chromosomic abnormalities that could pose risk of death; persistent physiological instability including hypotension, bradycardia, seizures, unresolved pneumothorax, or pneumomediastinum; diagnosis of congenital infection or chorioamnionitis at birth.

An independent Patient Safety and Data Committee was formed for external study monitoring.

The study was approved by the Research Ethics Committees of all participating units, as well as by the National Research Ethics Commission (CONEP), under register CEP: 292/02, process number: 25000.D02529/2003-40, CONEP opinion: 620/2003.

### Randomization and formation of the study groups

The animal-derived surfactants available in the country are Survanta^®^ (Abbott Laboratories, Ohio, USA) and Curosurf^®^*(Chiesi Farmaceutici, *Parma, Italy*).*The newborns were randomized to receive either the Butantan surfactant, comprising the Butantan group, or the commercial surfactant routinely used in the participating institution (Survanta^®^ or Curosurf^®^), comprising the control group. Randomization was stratified separately according to the birth weight range (501-750g, 751-1,000g, and 1,001-1,500g) and to each participating study center.

The patients were randomized using blocks of four, thus allowing six draw combinations (AABB; ABAB; ABBA; BABA; BAAB and BBAA). Thus, each number drawn by lot corresponded to four children. This method has two advantages: it allows that, at every four newborns included in the study, there is always the same number of participants receiving treatment A or B; and it makes it difficult to predict the treatment to be administered to the next child included in the study, especially when performed separately for each stratum. One-digit numbers were repeatedly drawn by lot until the sample size required for each stratum was obtained.

The randomization process was performed by professionals from the Department of Epidemiology of the Public Health College,*Universidade de São Paulo*. The treatment assigned by randomization was noted down in a sealed opaque envelope. To ensure that the surfactant used in each treatment was not identified, the envelope was opened, the medication prepared, and surfactant was delivered in a sealed syringe to the team directly in charge of drug administration by persons not involved in the study (support pharmacist or nurse). All surfactants were kept at a temperature of approximately 4°C. The physicians in charge of the newborns remained blind to the type of surfactant used in each treatment until study completion.

### Treatment strategy and post-treatment controls

A 100mg dose of phospholipids/kg was used for all newborns, in all treatments. Prior to administration, suctioning was performed only if necessary. After treatment, suctioning was performed again, observing a minimum 1-hour interval between administration and suctioning, or anytime if endotracheal tube obstruction was suspected. Treatment was given if the newborns did not present with physiological instability (presence of hypotension, bradycardia, seizure, unresolved pneumothorax, or pneumomediastinum), with the administration of a single dose, via endotracheal tube, at a constant speed, avoiding medication reflux. In each participating center, the treatment provided to newborns of the two study groups was identical.

Each newborn could receive two adittional surfactant doses at most, and the retreatment criterium was established when the PaO_2_/FiO_2 _ratio was ≤175, without any other cause that could explain worsening of the respiratory function (for instance: pneumothorax and pulmonary hypertension). Retreament was performed after a minimum period of 6 hours following the previous dose and up to a maxium limit of 48 hours of life, using the same dose and administration technique.

Blood samples were drawn for arterial blood gas immediately before and 1 and 6 hours after administration of the first surfactant dose, for the calculation of the oxygenation index (OI), using the formula OI=(MAP x FiO_2_)/PaO_2_ x 100, where MAP corresponds to the mean airway pressure. The ventilator settings were noted down immediately before and at 1, 6, 12, 18, 24, 30, 36, 42 and 48 hours after treatment, for the calculation of MAP values for each study group.

### Definition of primary outcome measures

The primary outcome measures for this study were mortality at 72 hours and 28 days of life. The secondary outcome measures included complications typically associated with prematurity and mechanical ventilation.

## Statistical analysis

Continuous variables were analyzed using the Student’s t or Mann-Whitney test, as appropriate. Qualitative variables were analyzed using the χ^2^ or Fischer’s exact test, as appropriate. The sample size calculated to detect an increase in overall mortality from 23.0% to 33.0%, as was observed in the 41 Brazilian maternity hospitals participating in the Analysis and Intervention Project for the Improvement of Neonatal Care in Brazil, from 2009 to 2011, considering a significance level of 0.05 and a power of test of 0.8, was 316 newborns. The significance level was set at p≤0.05. The Stata – Data Analysis and Statistical Software version 10.0 (StataCorp, USA) was used for data analysis.

## RESULTS

**Figure 1:**
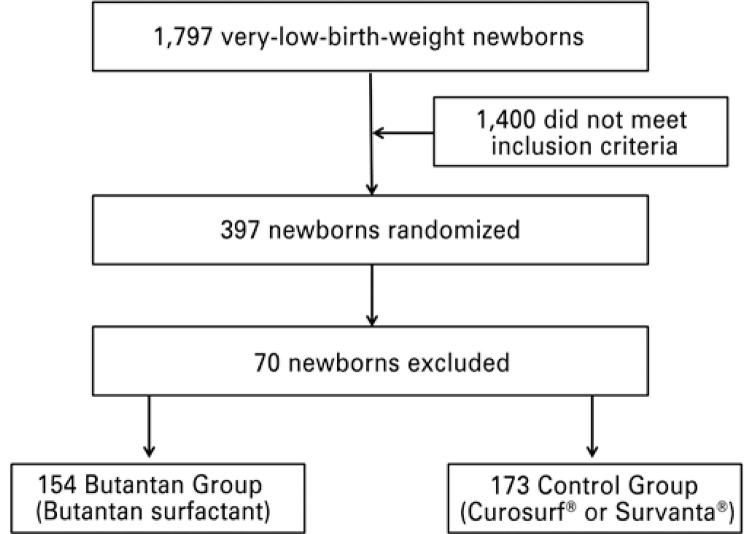
Patient selection flow chart

A total of 1,797 very-low-birth-weight newborns were enrolled during the study period; 397 of them were randomized (Figure 1). Of these, 70 newborns were excluded for: not meeting all the inclusion criteria; retreatment after more than 48 hours of life; newborn randomized, but not treated with surfactant; presence of chorioamnionitis; administration of more than three surfactant doses; breach of confidentiality on the surfactant to be administered; failure to follow the surfactant randomization; diagnosis of major malformation; birth outside the participating institution; clinical center excluded from the study; incomplete data recording; lack of written informed consent; lack of pre-treatment blood gas; hemodinamically unstable newborn; data recorded only after the second surfactant dose; newborn randomized, but not on mechanical ventilation; and lack of surfactant in the institution after administration of the second surfactant dose. Of the remaining 327 newborns, 154 were randomized to the Butantan group, and 173 to the control group (Figure 1). The 1,400 non-randomized newbords did not fulfill all inclusion criteria. Main peri and postnatal demographic characteristcs of 327 newborns are showed in table 1. The mean birth weight (990±245g *versus* 996±235g) and gestational age (28.0±2.1 weeks *versus* 28.1±2.2 weeks) were similar for the control and Butantan groups, respectively. No differences were observed regarding sex, prenatal use of corticosteroid, mode of delivery, and classification according to weight and gestational age. Higher 1- and 5-minute Apgar scores were observed among newborns of the control group. The birth weight of approximately half of the children randomized to both groups was between 1,000 and 1,500g.

**Table 1:** Peri- and postnatal characteristics of the 327 newborns studied

Characteristics	Control group (n=173)	Butantan group (n=154)	p value
Birth weight, g	990.65±245.70	996.01±235.84	0.84
Gestational age, weeks	28.03±2.07	28.14±2.16	0.64
Sex, n (%)			
Male	86 (49.71)	84 (54.55)	0.38
Female	87 (50.29)	70 (45.45)	0.38
Prenatal corticosteroid, n (%)	94 (54.34)	79 (51.30)	0.58
Vaginal birth, n (%)	66 (38.15)	61 (39.66)	0.79
C-section birth, n (%)	107 (61.85)	93 (60.93)	0.79
1’ Apgar	5.2±2.4	4.5±2.3	0.006
5’ Apgar	7.9±1.5	7.4±1.7	0.02
AGI, n (%)	125 (72.25 )	121 (78.57)	0.19
SGA, n (%)	48 (27.75)	33 (21.43)	0.19
LGA, n (%)	0	0	-
BW <750g, n (%)	32 (18.5)	23 (14.9)	0.69
BW ≥750g and <1,000g, n (%)	61 (35.26)	57 (37.01)	0.69
BW ≥1,000g and <1,500g, n (%)	80 (46.24)	74 (48.05)	0.69

BW, Apagar score, and gestational age values are expressed as mean ± standard deviation. Continuous variables were analyzed using the Student’s t or Mann-Whitney test, as appropriate. Qualitative variables were analyzed using the χ^2^ or Fischer’s exact test, as appropriate. AGA: appropriate for gestational age; SGA: small for gestational age; LGA: large for gestational age; BW: birth weight.

Butantan group newborns required more surfactant doses (mean ± standard deviation) in comparison to those of the control group (2.0±0.8 *versus* 1.5±0.7, respectively; p<0,001). No difference was found between the two groups analyzed in relation to the moment (hours of life) at which the first surfactant dose was administered: control group, at 5.35 hours (median), and Butantan group, at 4,87 hours (median) (p=0,30).

### Primary outcome measures

No diferences were observed in relation to mortality between the Butantan and control groups, both at 72 hours (14.19%*versus *14.12%; p=0.98) and at 28 days of life (39.86%*versus*33.33%; p=0.24). Also, no differences were observed between the groups in relation to mortality, when analyzed by birth weight range, including birth weight <750g; birth weight between 750 and 999g; and birth weight between 1,000 and 1,499g (Tables 2 and 3).

**Table 2:** Overall mortality and by birth weight range within the first 72 hours after the first surfactant dose

	Control group n (%)	Butantan group n (%)	p value
Overall mortality	24 (14.12)	22 (14.19 )	0.98
BW <750g (n=55)	11 (35.48 )	10 (39.13 )	0.64
BW ≥750g and <1,000g (n=115)	8 (13.56 )	8 (14.29 )	1.0
BW ≥1,000g and <1,500g (n=151)	5 (6.36 )	4 (5.48 )	1.0

Continuous variables were analyzed using the Student’s t or Mann-Whitney test, as appropriate. Qualitative variables were analyzed using the χ^2^ or Fischer’s exact test, as appropriate. BW: birth weight.

**Table 3:** Overall mortality and by birth weight range on the 28th day of life

Secondary variables	Control group n (%)	Butantan group n (%)	p value
Overall mortality	55 (33.33)	57 (39.86)	0.24
BW <750g (n=53)	21 (67.74)	15 (68.18)	0.97
BW ≥750g and <1,000g (n=109)	23 (39.66)	26 (50.98)	0.24
BW ≥1,000g and <1,500g (n=146)	11 (14.47)	16 (22.86)	0.19

Continuous variables were analyzed using the Student’s t or Mann-Whitney test, as appropriate. Qualitative variables were analyzed using the χ^2^ or Fischer’s exact test, as appropriate. BW: birth weight.

### Secondary outcome measures

No differences were observed among newborns of the control and Butantan groups as regards the secondary variables analyzed on the 28th day of life (Table 4), except for greater need for supplemental oxygen (45.28%*versus*57.04%; p=0.05), and higher frequency of the diagnosis of persistent ductus arteriosus (31.45%*versus*44.93%; p=0.02) and of interstitial pulmonary emphysema (7.64%*versus*17.04%; p=0.01) in Butantan group newborns. The frequency of pulmonary hemorrhage was similar in both groups (12.58%*versus*11.59%; p=0.80).

**Table 4:** Frequency of secondary variables analysed on the 28th day of life

Secondary variables	Control group n (%)	Butantan group n (%)	p value
Use of oxygen at 28 days	72 (45.28)	77 (57.04)	0.05
Radiography consistent with BPD	52 (65.00)	57 (72.15)	0.33
Intracranial hemorrhage (total)	39 (24.53)	43 (31.16)	0.20
Grade I	17 (43.59)	17 (39.53)	0.94
Grade II	5 (12.82)	7 (16.28)	
Grade III	10 (25.64)	10 (23.26)	
Grade IV	7 (17.95)	9 (20.93)	
PDA (n=297)	50 (31.45)	62 (44.93)	0.02
Early sepsis (n=297)	58 (36.48)	50 (36.23)	0.97
Late sepsis (n=293)	59 (37.58)	51 (37.50)	0.99
Pulmonary hemorrhage (n=297)	20 (12.58)	16 (11.59)	0.80
Pneumothorax (n=297)	13 (8.18)	12 (8.70)	0.87
Interstitial emphysema (n=292)	12 (7.64)	23(17.04)	0.01
Necrotizing enterocolitis (n=297)	11 (6.92)	11 (7.97)	0.83
Mechanical ventilation time (days)	8	12	0.06
Total oxygen therapy time (days)	17	22	0.35

Continuous variables were analyzed using the Student’s t or Mann-Whitney test, as appropriate. Qualitative variables were analyzed using the χ^2^ or Fischer’s exact test, as appropriate. BPD: bronchopulmonary displasia; PDA: persistent ductus arteriosus.

**Figure 2:**
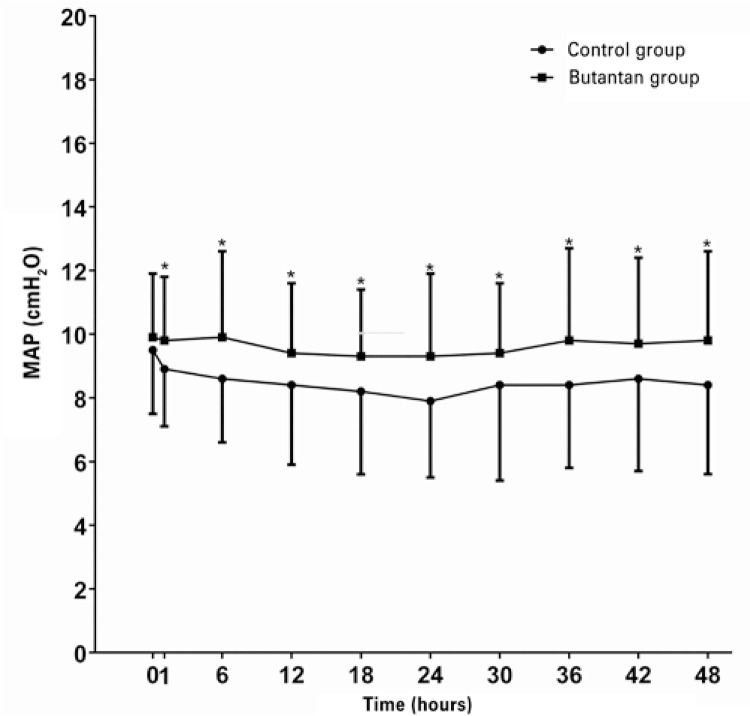
Mean airway pressure values, pre- and post-treatment, in both study groups

Reductions in both OI (Table 5) and MAP (Figure 2) values were observed in the two groups after treatment with exogenous surfactant. However, the magnitude of decrease was greater in the newborns treated with Survanta^®^ or Curosurf^®^, in relation to those treated with the Butantan surfactant.

**Table 5:** Oxygenation index values prior to treatment and 1 and 6 hours after (values in percentiles)

Percentile	p value	10		25		50		75		90	
Group		CG	BG	CG	BG	CG	BG	CG	BG	CG	BG
Pre-treatment	0.92	5.4	5.4	7.2	6.7	10.7	10.9	16.2	17.3	24.5	25.6
1 hour after	<0.001	2.4	2.7	3.0	4.6	4.6	7.8	7.2	12.7	13.2	20.1
6 hours after	<0.001	2.0	2.3	2.6	4.0	4.0	6.8	6.4	10.1	12.0	19.3

Continuous variables were analyzed using the Student’s t or Mann-Whitney test, as appropriate. Qualitative variables were analyzed using the χ^2^ or Fischer’s exact test, as appropriate. CG: control group; BG: Butantan group. Values expressed in percentiles.

In relation to the complications observed during surfactant administration, we observed that less children of the Butantan group showed bradycardia in relation to those of the control group (8.3%*versus *36.7%; p=0.002); both groups showed a similar incidence of surfactant reflux up the endotracheal tube (83.3%*versus*76.7%; p=0.470).

## DISCUSSION

The main objective of this multicenter study was to verify the efficacy and safety of a new surfactant manufactured by *Instituto Butantan* in relation to similar products commercially available in Brazil. Mortality rates at 72 hours and on the 28th day of life were similar to those observed with the use of two animal-derived surfactants used in our setting. These observations show that the major benefit of using exogenous surfactants for the treatment of newborns with RDS, *i.e.* , mortality reduction, was obtained. This is important if we consider that, in several public maternity hospitals in our country, exogenous surfactant is not routinely available for clinical use because of its high cost. Equally important is the finding of a similar rate of the main diseases related to prematurity in both study groups, including bronchopulmonary dysplasia, grades III and IV intracranial hemorrhage, and necrotizing enterocolitis. Although the Butantan group had had a lower 5-minute Apgar score (7.9±1.5 *versus* 7.4±1.7; p=0.006), the magnitude of the difference had no clinical significance.

Although the incidence of pneumothorax had been very similar in both study groups, children treated with the Butantan surfactant showed a higher incidence of pulmonary interstitial emphysema. Both groups showed a quite higher overall incidence of extrapulmonary air than that reported, both with the use of lucinactant (a synthetical surfactant awaiting Food and Drug Administration – FDA approval) and of poractant alfa (9.2% and 7.3%, respectively).^(13)^ Immediate response to surfactant therapy was more evident in the control group, as demonstrated by a more marked reduction in the mean airway pressure and better OI values after treatment. However, when the variations in these parameteres are analyzed in both study groups, we verify that they were not associated with clinical differences between the groups, and this is demonstrated by a similar mechanical ventilation time, total oxygen time, and need for supplemental oxygen at 28 days of life, in addition to a similar mortality rate at 72 hours and 28 days of life, and incidence of pulmonary hemorrhage and pneumothorax.

For approximately two decades, the international literature has been discussing about the best way to evaluate whether a surfactant works adequately and how the efficacy of two surfactants may be compared in the treatment of a determined respiratory disease. Based on the evidences published, it is admitted that the acute effects of surfactants on the lungs (as assessed by oxygenation parameters) do not clearly predict their efficacy, and are less important than their influence on mortality associated with respiratory failure.^(14-17)^ The first evidence of this fact is in studies comparing animal-derived to synthetic surfactants, in which it is clear that the surfactant used has its function improved after exposure to the preterm lung and modification within the pneumocyte II, being further released to the alveoli.^(14)^ Thus, the assessment of mortality assicated with respiratory failure is the most important parameter in the evaluation of the therapeutic efficacy of a surfactant, in comparison to the evaluation of oxygenation itself. The second evidence is shown with the use of exogenous surfactants in the treatment of meconium aspiration syndrome in newborns.^(18,19)^ In this disease, the international literature shows that exogenous surfactante therapy improves oxygenation in the short term. However, despite oxygenation improvement, clinical studies do not confirm a mortality reduction in meconium aspiration syndrome with exogenous surfactant therapy; thus, the use of surfactant in this condition is controversial and lacks consensus.^(20)^ This stresses the superiority of the assessment of mortality in the analysis of the therapeutic efficacy of surfactants, in relation to its other effects on oxygenation.

In addition to the analysis of efficacy, it is also necessary to evaluate the safety of this new drug on the short (moment of administration) and long term (events that may arise as a result of its use). In this study, the safety of both groups was analyzed at the moment of administration (bradycardia and surfactant reflux up the endotracheal tube) and as regards the occurence of possible adverse effects associated with surfactants. The results obtained showed that, during surfactant administration, there was a higher incidence of surfactant reflux up the endotracheal tube among newborns of the Butantan group, and of bradycardia after surfactant administration among newborns of the control group. In relation to the adverse effects associated with the use of surfactants, we point out the occurrence of pulmonary hemorrhage, which is described in the international literature as the most severe event associated with this therapy.^(21)^ The results obtained in this study showed that the incidence of pulmonary hemorrhage was similar in both groups analyzed.

As regards the surfactant dose used in this study, a certain number of clinical and pharmacokinetic studies show that higher doses are more efficient.^(22)^ The choice to use the 100mg/kg dose was based on the fact that, in RDS, serum-derived protein inhibitors and inflammatory mediators that inactivate the surfactant system accumulate progressively, and treatment made in the initial phase is effective with the 100mg/kg dose.^(23)^

Several studies have been conducted to compare synthetic (with no protein in their composition) to natural (bovine- or porcine-derived) surfactants, with better results achieved by animal-derived in relation to synthetic surfactants. Corroborating our findings, comparisons between different animal-derived surfactants (with apoproteins B and C in their composition) did not show different results in relation to mortality and the main morbidities related to prematurity, including mechanical ventilation settings, incidence of pneumothorax, insterstitial emphysema, intracranial hemorrage, and persistent ductus arteriosus, when compared at the same treatment dose.^(24,25), 26)^

## CONCLUSION

The new surfactant developed for the treatment of respiratory distress syndrome in newborns is efficient and safe, and this was demonstrated by a similar neonatal mortality rate and incidence of pulmonary hemorrhage and pneumothorax in both study groups.
