# Supplementary material for: A multicenter, randomized, double-blind trial of a new porcine surfactant in premature infants with respiratory distress syndrome
Source: Einstein (Sao Paulo). 2014 Oct-Dec;12(4):397–404. doi: 10.1590/S1679-45082014AO3095 (PMC4879903; doi:10.1590/S1679-45082014AO3095)
Supplement: Translation

# Translation: Ensaio clínico multicêntrico duplo-cego randomizado com um novo surfactante de origem porcina em prematuros com síndrome do desconforto respiratório

**Authors:** Celso Moura Rebello, Alexander Roberto Precioso, Renata Suman Mascaretti, Grupo Colaborativo do Estudo Brasileiro Multicêntrico de Surfactante

**Affiliations:** 1 Hospital Israelita Albert Einstein, São Paulo, SP, Brasil.; 2 Instituto da Criança, Hospital das Clínicas, Faculdade de Medicina, Universidade de São Paulo, São Paulo, SP, Brasil.; 3 Maternidade Pro Matre Paulista, São Paulo, SP, Brasil.

**Keywords:** Surfactantes pulmonares/uso terapêutico, Recém-nascido de muito baixo peso, Recém-nascido, Prematuro, Síndrome do desconforto respiratório do recém-nascido/quimioterapia

## Abstract

**Objetivo:**

Comparar a eficácia e a segurança de um novo surfactante pulmonar de origem porcina, desenvolvido pelo Instituto Butantan, com os surfactantes de origem animal disponíveis no país, em relação à mortalidade neonatal e às principais complicações da prematuridade, em prematuros com peso de nascimento até 1500g e diagnóstico de síndrome do desconforto respiratório.

**Métodos:**

Recém-nascidos com diagnóstico de síndrome do desconforto respiratório foram randomizados para receber surfactante Butantan (Grupo Butantan) ou um dos seguintes surfactantes: Survanta^®^ou Curosurf^®^. Os recém-nascidos que receberam Survanta^®^ou Curosurf^®^formaram o Grupo Controle. Foram definidas, como variáveis primárias, as mortalidades com 72 horas e 28 dias de vida e, como variáveis secundárias, as principais complicações típicas da prematuridade, avaliadas no 28^O^dia de vida.

**Resultados:**

Não foram observadas diferenças em relação ao peso de nascimento, idade gestacional, sexo e corticoide pré-natal, assim como em relação à mortalidade dos recém-nascidos dos Grupos Butantan (n=154) e Controle (n=173), tanto com 72 horas (14,19% *versus* 14,12%; p=0,98) como em 28 dias de vida (39,86% *versus* 33,33%; p=0,24). Foram observados maiores valores do boletim de Apgar de 1 e de 5 minutos entre os recém-nascidos do Grupo Controle. Os grupos não diferiram em relação às variáveis secundárias, exceto por uma maior necessidade de uso de oxigênio e de enfisema pulmonar intersticial no Grupo Butantan.

**Conclusão:**

As taxas de mortalidade com 72 horas e 28 dias de vida, assim como a incidência das principais complicações da prematuridade, foram comparáveis àquelas encontradas com os surfactantes de origem animal comercialmente disponíveis no Brasil, mostrando a eficácia e a segurança do novo surfactante no tratamento da síndrome do desconforto respiratório em recém-nascidos.

## INTRODUÇÃO

A síndrome do desconforto respiratório (SDR) neonatal é causada primariamente por uma deficiência qualitativa e quantitativa de surfactante ao nascimento, sendo Avery e Mead os responsáveis pelos primeiros estudos que relacionaram a deficiência de surfactante à essa doença.^(1)^

O primeiro estudo experimental que demonstrou a ação benéfica da reposição de surfactante foi realizado no início da década de 1970 por Enhornig e Robertson,^(2)^por meio da instilação de surfactante natural obtido dos pulmões de coelhos adultos na traqueia de coelhos prematuros. Trabalhos subsequentes foram realizados tanto para comprovar sua eficácia quanto para aperfeiçoar sua extração e purificação. Um dos pioneiros nos estudos clínicos foi Fujiwara et al.,^(3)^que comprovaram, em um estudo não controlado, a melhora da oxigenação após a administração do surfactante, em dez recém-nascidos prematuros.

No início dos anos 1990, o surfactante passou a ser utilizado como rotina em vários centros de cuidados intensivos neonatais. Desde então, tem-se procurado a obtenção do “surfactante ideal” para o uso rotineiro, caracterizado por eficácia comprovada, segurança, boa resistência à inativação por proteínas plasmáticas e baixo custo. Ao surfactante, são atribuídas as seguintes funções: evitar o colabamento alveolar no final da expiração, diminuir secundariamente o edema alveolar e apresentar ações relacionadas à imunidade.^(4)^

A terapêutica com o surfactante exógeno permitiu uma redução das taxas de mortalidade neonatal entre os prematuros extremos, com impacto inclusive sobre a mortalidade infantil nos países desenvolvidos. Uma limitação para seu uso em larga escala, em prematuros e também em crianças maiores e adultos, é seu alto custo. Dessa forma, o Instituto Butantan, localizado na cidade de São Paulo (SP), visando atenuar esse problema a nível nacional, desenvolveu um novo método de extração e purificação do surfactante natural de origem porcina, obtendo um novo produto, com custo de produção reduzido em relação aos produtos importados.^(5,6)^

Esse novo processo se baseou na maceração de pulmão de suínos, seguida de extração orgânica acoplada à adsorsão em dietilaminoetil celulose (DEAE-C). Essa tecnologia teve a vantagem de reduzir os custos de produção, não apenas por não utilizar grandes ultracentrífugas refrigeradas de escala industrial, mas também pelo reaproveitamento dos reagentes utilizados nas várias etapas da extração. Sua composição química é bastante semelhante à de outros surfactantes de origem animal, sendo o conteúdo lipídico representado pela fosfatidilcolina (76%), fosfatidiletanolamina (6 a 8%), fosfatidilglicerol (6%), fosfatidilinositol (6%) e esfingomielina (4 a 6%), além das proteínas B e C do surfactante (5,6%).^(5)^Esse produto teve sua eficácia comprovada*in vivo*, utilizando-se um modelo experimental de SDR.^(7)^Em vista do risco teórico de imunogenicidade às proteínas presentes no surfactante pulmonar,^(8,8)^a resposta imunológica à exposição ao novo produto foi pesquisada, sendo demonstrado, em modelo animal, que o surfactante produzido pelo Instituto Butantan não esteve associado à ocorrência de manifestações clínicas que inviabilizassem seu uso em recém-nascidos prematuros com SDR.^(10)^

O novo surfactante também foi utilizado para o tratamento da síndrome de aspiração meconial em modelo animal.^(11,12)^Os resultados desses estudos demonstraram que os animais tratados com o surfactante produzido pelo Instituto Butantan apresentaram melhora significativa da mecânica pulmonar, dos volumes pulmonares e dos padrões histopatológicos de aeração alveolar.^(11,12)^Com base nesses estudos, foi aventada a hipótese de que o surfactante produzido pelo Instituto Butantan é tão eficaz e seguro quanto os surfactantes disponíveis comercialmente no Brasil para o tratamento da SDR neonatal.

## OBJETIVO

Avaliar a eficácia e segurança de um novo surfactante pulmonar de origem porcina, desenvolvido pelo Instituto Butantan, para o tratamento da síndrome do desconforto respiratório neonatal, quando comparado aos surfactantes de origem animal disponíveis no país, particularmente em relação à mortalidade neonatal, à incidência de hemorragia pulmonar e à de pneumotórax.

## MÉTODOS

### População do estudo

Foi realizado um estudo multicêntrico, prospectivo, randomizado, incluindo 19 unidades de terapia intensiva neonatal distribuídas nos Estados de São Paulo (6 centros), Rio de Janeiro (3 centros), Minas Gerais (3 centros), Sergipe (2 centros), Rio Grande do Sul (2 centros), Pernambuco (1 centro), Distrito Federal (1 centro) e Maranhão (1 centro), no período de abril de 2005 a agosto de 2007. As gestantes com alto risco de parto prematuro (acima de 24 semanas de gestação e peso de nascimento estimado entre 501 e 1.500g) foram abordadas em relação à participação no estudo. Se os critérios de inclusão fossem atingidos e o consentimento livre e esclarecido fosse obtido, o recém-nascido era considerado elegível para randomização.

Foram critérios de inclusão: recém-nascidos prematuros nascidos nos centros envolvidos nesse estudo; com peso de nascimento entre 501 e 1.500g; idade pós-natal ≤24 horas de vida; em ventilação mecânica; com relação entre pressão arterial de oxigênio e fração inspirada de oxigênio (PaO_2_/FiO_2_) ≤175; diagnóstico clínico e radiológico de SDR; e consentimento livre e esclarecido assinado pelos pais ou responsável legal.

Os critérios de exclusão foram: tratamento prévio com outro tipo de surfactante; recém-nascidos com malformações congênitas maiores ou alterações cromossômicas previamente diagnosticadas que acarretassem em risco de vida; instabilidade fisiológica persistente, incluindo hipotensão, bradicardia, convulsão, pneumotórax não resolvido ou pneumomediastino; Diagnóstico de infeção congênita ou ovular ao nascimento.

Um Comitê de Dados e de Segurança do Paciente independente foi constituído com a finalidade de monitorização externa do estudo.

O estudo foi aprovado pelos Comitês de Ética em Pesquisa de todas as unidades participantes, assim como pela Comissão Nacional de Ética em Pesquisa (CONEP), com registro CEP: 292/02, processo número: 25000.D02529/2003-40, parecer CONEP: 620/2003.

### Randomização e formação dos grupos de estudo

Os surfactantes de origem animal disponíveis no país são o Survanta^®^(*Abbott Laboratories,* Ohio, Estados Unidos) e Curosurf^®^(*Chiesi Farmaceutici*, Parma, Itália). Os recém-nascidos foram randomizados para receber o surfactante Butantan, constituindo o Grupo Butantan, ou o surfactante comercial de uso de rotina da instituição participante (Survanta^®^ou Curosurf^®^), constituindo, assim, o Grupo Controle. A randomização foi estratificada separadamente de acordo com a faixa de peso de nascimento (501-750g, 751-1.000g, e 1.001-1.500g) e com cada centro participante do estudo.

O método utilizado para a randomização das crianças foi o de blocos de quatro, o que possibilita seis combinações de sorteio (AABB; ABAB; ABBA; BABA; BAAB e BBAA). Assim, cada número sorteado correspondeu a quatro crianças. Esse método apresenta duas vantagens: permite que, a cada quatro recém-nascidos que entram no estudo, haja sempre uma mesma quantidade de participantes submetidos ao tratamento A ou B; e dificulta a previsão do tratamento a ser administrado para a próxima criança incluída no estudo, especialmente quando realizado separadamente para cada estrato. Foram sorteados, repetidamente, números de um algarismo, até se obter o tamanho amostral necessário para cada estrato.

O processo de randomização foi realizado pelos profissionais do Departamento de Epidemiologia da Faculdade de Saúde Pública da Universidade de São Paulo. O tratamento assinalado pela randomização foi anotado em um envelope opaco e lacrado. Para assegurar a não identificação do surfactante usado em cada tratamento, a abertura do envelope, o preparo da medicação e a entrega do surfactante em seringa lacrada para equipe responsável diretamente pela administração foi feita por pessoas não envolvidas no estudo (farmacêutico ou uma enfermeira de apoio). Todos os surfactantes foram estocados a uma temperatura de cerca de 4°C. Os médicos responsáveis pelos recém-nascidos permaneceram sem informação em relação ao tipo de surfactante utilizado em cada tratamento até o final do estudo.

### Estratégia de tratamento e controles após o tratamento

Foi utilizada a dose de 100mg de fosfolípides/kg para todos os recém-nascidos, em todos os tratamentos. Antes da administração, os recém-nascidos foram aspirados apenas se necessário. Após o tratamento, novas aspirações foram realizadas, respeitando-se um intervalo mínimo de 1 hora entre a administração e a aspiração, ou a qualquer momento se houvesse a hipótese de obstrução da cânula endotraqueal. O tratamento foi realizado se os recém-nascidos não apresentassem instabilidade fisiológica (presença de hipotensão, bradicardia, convulsão, pneumotórax não resolvido ou pneumomediastino), com administração em alíquota única, por cânula orotraqueal, em velocidade constante, evitando-se refluxo da medicação. Em cada centro participante, os recém-nascidos dos dois grupos de estudo foram tratados de maneira idêntica.

Cada recém-nascido poderia receber no máximo duas doses adicionais de surfactante, sendo que o critério de retratamento foi estabelecido quando a relação PaO_2_/FiO_2_fosse ≤175, sem outra causa que explicasse a piora da função respiratória (por exemplo: pneumotórax e hipertensão pulmonar). O retratamento foi realizado após um período mínimo de 6 horas da dose anterior e até um limite máximo de 48 horas de vida, com igual dose e técnica de administração.

Uma gasometria arterial foi coletada imediatamente antes e 1 e 6 horas após a administração da primeira dose do surfactante, para o cálculo do índice de oxigenação (IO), por meio da fórmula IO=(MAP x FiO_2_)/ PaO_2_x 100, sendo que MAP corresponde à pressão média das vias aéreas. Os parâmetros do ventilador foram anotados imediatamente antes e com 1, 6, 12, 18, 24, 30, 36, 42 e 48 horas após o tratamento, para cálculo os valores da MAP para cada grupo de estudo.

### Definição das variáveis primárias

As variáveis primárias para este estudo foram a mortalidade com 72 horas e com 28 dias de vida. As variáveis secundárias incluíram as complicações tipicamente associadas à prematuridade e à ventilação mecânica.

### Análise estatística

As variáveis contínuas foram analisadas pelo teste*t* de Student ou de Mann-Whitney, conforme o mais indicado. As variáveis qualitativas foram analisadas pelo teste do χ^2^ou exato de Fischer, conforme o mais apropriado. O cálculo amostral para se detectar um aumento na mortalidade geral de 23,0% para 33,0%, conforme observado nas 41 maternidades brasileiras que participam do Projeto Análise e Intervenção para Melhoria do Cuidado Neonatal no Brasil, de 2009 a 2011, considerando-se um nível de significância de 0,05 e um poder de teste de 0,8, foi de 316 recém-nascidos. O nível de significância adotado foi um valor de p≤0,05. Foi utilizado para análise o *software Stata – Data Analysis and Statistical Software, versão 10.0 (StataCorp,* Estados Unidos).

## RESULTADOS

Um total de 1.797 recém-nascidos de muito baixo peso foram admitidos no período do estudo, dos quais 397 foram randomizado (Figura 1). Destes, 70 recém-nascidos foram excluídos. Dentre as causas das exclusões, estiveram o não cumprimento de todos os critérios de inclusão; o retratamento com mais de 48 horas de vida; recém-nascido randomizado, mas sem tratamento com surfactante; presença de corioamnionite; administração de mais de três doses de surfactante; a quebra de sigilo sobre o surfactante a ser administrado; o não seguimento da randomização do surfactante; o diagnóstico de malformação maior; nascimento fora da instituição participante; centro clínico excluído do estudo; o preenchimento incompleto dos dados; a não obtenção do Termo de Consentimento Livre e Esclarecido; ausência de gasometria pré-tratamento; recém-nascido com instabilidade hemodinâmica; dados registrados apenas a partir da segunda dose de surfactante; recém-nascido randomizado, porém fora de ventilação mecânica; e a falta de surfactante na instituição após a administração da segunda dose de surfactante. Dos 327 recém-nascidos restantes, 154 foram randomizados no Grupo Butantan e 173 no Grupo Controle (Figura 1). Os 1.400 recém-nascidos que não randomizados não preencheram todos os critérios de inclusão. As principais características demográficas peri e pós-natais dos 327 recém-nascidos analisados são mostradas na tabela 1 . Os valores médios de peso de nascimento (990±245g *versus* 996±235g) e idade gestacional (28,0±2,1 semanas *versus* 28,1±2,2 semanas) foram similares para os Grupos Controle e Butantan, respectivamente. Não foram observadas diferenças em relação ao sexo, ao uso de corticoide pré-natal, à via de parto, à classificação segundo o peso e a idade gestacional. Foram observados maiores valores do boletim de Apgar de 1 e de 5 minutos entre os recém-nascidos do Grupo Controle. Cerca de metade das crianças randomizadas em ambos os grupos tiveram peso de nascimento entre 1.000 e 1.500g.

**Figura 1:** Fluxograma da seleção dos pacientes

**Tabela 1:** Características peri e pós-natais dos 327 recém-nascidos analisados

Características	Grupo Controle (n=173)	Grupo Butantan (n=154)	Valor de p
Peso de nascimento, g	990,65±245,70	996,01±235,84	0,84
Idade gestacional, semanas	28,03±2,07	28,14±2,16	0,64
Sexo, n (%)			
Masculino	86 (49,71)	84 (54,55)	0,38
Feminino	87 (50,29)	70 (45,45)	0,38
Corticoide pré-natal, n (%)	94 (54,34)	79 (51,30)	0,58
Parto vaginal, n (%)	66 (38,15)	61 (39,66)	0,79
Parto cesáreo, n (%)	107 (61,85)	93 (60,93)	0,79
Apgar 1’	5,2±2,4	4,5±2,3	0,006
Apgar 5’	7,9±1,5	7,4±1,7	0,02
AIG, n (%)	125 (72,25 )	121 (78,57)	0,19
PIG, n (%)	48 (27,75)	33 (21,43)	0,19
GIG, n (%)	0	0	-
PN <750g, n (%)	32 (18,5)	23 (14,9)	0,69
PN ≥750g e <1.000g, n (%)	61 (35,26)	57 (37,01)	0,69
PN ≥1.000g e <1.500g, n (%)	80 (46,24)	74 (48,05)	0,69

Os resultados de PN, Apgar e idade gestacional são apresentados em média ± desvio padrão. Variáveis contínuas foram analisadas pelo teste *t* de Student ou de Mann-Whitney, conforme o mais indicado. Variáveis qualitativas analisadas pelo teste do χ^2^ou exato de Fischer, conforme o mais apropriado. AIG: adequados para a idade gestacional; PIG: pequenos para a idade gestacional; GIG: grandes para a idade gestacional; PN: peso de nascimento.

Os recém-nascidos do Grupo Butantan necessitaram de mais doses de surfactante (média ± desvio padrão) quando comparados aos do Grupo Controle (2,0±0,8*versus*1,5±0,7; p<0,001). Não houve diferença em relação ao momento (horas de vida) em que a primeira dose de surfactante foi administrada nos dois grupos analisados: Grupo Controle, com 5,35 horas (mediana), e Grupo Butantan, com 4,87 horas (mediana) (p=0,30).

### Desfechos primários

Não foram observadas diferenças em relação à mortalidade nos Grupos Butantan e Controle, tanto com 72 horas (14,19%*versus* 14,12%; p=0,98) como com 28 dias de vida (39,86%*versus* 33,33%; p=0,24). Também não foram observadas diferenças em relação à mortalidade em ambos os grupos quando analisadas por faixas de peso de nascimento, incluindo peso de nascimento <750g; peso de nascimento entre 750 e 999g, e peso de nascimento entre 1.000 e 1.499g ( Tabelas 2 e 3).

**Tabela 2:** Mortalidade geral e por faixa de peso dentro das primeiras 72 horas após a primeira dose de surfactante

	Grupo Controle n (%)	Grupo Butantan n (%)	Valor de p
Mortalidade geral	24 (14,12)	22 (14,19 )	0,98
PN <750g (n=55)	11(35,48 )	10 (39,13 )	0,64
PN ≥750g e <1.000g (n=115)	8 (13,56 )	8 (14,29 )	1,0
PN ≥1000g e <1.500g (n=151)	5 (6,36 )	4 (5,48 )	1,0

Variáveis contínuas foram analisadas pelo teste *t* de Student ou de Mann-Whitney, conforme o mais indicado. Variáveis qualitativas analisadas pelo teste do χ^2^ou exato de Fischer, conforme o mais apropriado. PN: peso de nascimento.

**Tabela 3:** Mortalidade geral e por faixa de peso no 28 o dia de vida

Variáveis secundárias	Grupo Controle n (%)	Grupo Butantan n (%)	Valor de p
Mortalidade geral	55 (33,33)	57 (39,86)	0,24
PN <750g (n=53)	21 (67,74)	15 (68,18)	0,97
PN ≥750g e <1.000g (n=109)	23 (39,66)	26 (50,98)	0,24
PN ≥1.000g e <1.500g (n=146)	11 (14,47)	16 (22,86)	0,19

Variáveis contínuas foram analisadas pelo teste *t* de Student ou de Mann-Whitney, conforme o mais indicado. Variáveis qualitativas analisadas pelo teste do χ^2^ou exato de Fischer, conforme o mais apropriado. PN: peso de nascimento.

### Desfechos secundários

Não foram observadas diferenças entre os recém-nascidos do Grupo Controle e os do Grupo Butantan em relação às variáveis secundárias analisadas no 28º dia de vida (Tabela 4), com exceção de uma maior necessidade de crianças em uso de oxigênio no Grupo Butantan (45,28% *versus* 57,04%; p=0,05), uma maior frequência de diagnóstico de persistência de canal arterial (31,45% *versus* 44,93%; p=0,02) e de enfisema pulmonar intersticial (7,64% *versus* 17,04%; p=0,01). A frequência de hemorragia pulmonar foi comparável em ambos os grupos (12,58% *versus* 11,59%; p=0,80).

**Tabela 4:** Frequência das variáveis secundárias analisadas no 28 o dia de vida

Variáveis secundárias	Grupo Controle n (%)	Grupo Butantan n (%)	Valor de p
Uso de oxigênio com 28 dias	72 (45,28)	77 (57,04)	0,05
Raios-x compatível com DBP	52 (65,00)	57 (72,15)	0,33
Hemorragia intracraniana (total)	39 (24,53)	43 (31,16)	0,20
Grau I	17 (43,59)	17 (39,53)	0,94
Grau II	5 (12,82)	7 (16,28)	
Grau III	10 (25,64)	10 (23,26)	
Grau IV	7 (17,95)	9 (20,93)	
PCA (n=297)	50 (31,45)	62 (44,93)	0,02
Sepse precoce (n=297)	58 (36,48)	50 (36,23)	0,97
Sepse tardia (n=293)	59 (37,58)	51 (37,50)	0,99
Hemorragia pulmonar (n=297)	20 (12,58)	16 (11,59)	0,80
Pneumotórax (n=297)	13 (8,18)	12 (8,70)	0,87
Enfisema intersticial (n=292)	12 (7,64)	23(17,04)	0,01
Enterocolite necrosante (n=297)	11 (6,92)	11 (7,97)	0,83
Tempo de ventilação mecânica (dias)	8	12	0,06
Tempo total de oxigenioterapia (dias)	17	22	0,35

Variáveis contínuas foram analisadas pelo teste *t* de Student ou de Mann-Whitney, conforme o mais indicado. Variáveis qualitativas analisadas pelo teste do χ^2^ou exato de Fischer, conforme o mais apropriado. DBP: displasia broncopulmonar; PCA: persistência do canal arterial.

Observou-se redução tanto dos valores do IO ( Tabela 5) como dos valores de MAP ( Figura 2) em ambos os grupos após o tratamento com surfactante exógeno, porém a magnitude da queda foi mais importante nos recém-nascidos tratados com Survanta^®^ou Curosurf^®^, em relação aos tratados com surfactante Butantan.

**Tabela 5:** Valores de índice de oxigenação antes do tratamento e 1 e 6 horas depois (valores em percentis)

Percentil	Valor de p	10		25		50		75		90	
Grupo		GC	GB	GC	GB	GC	GB	GC	GB	GC	GB
Pré tratamento	0,92	5,4	5,4	7,2	6,7	10,7	10,9	16,2	17,3	24,5	25,6
1 hora após	<0,001	2,4	2,7	3,0	4,6	4,6	7,8	7,2	12,7	13,2	20,1
6 horas após	<0,001	2,0	2,3	2,6	4,0	4,0	6,8	6,4	10,1	12,0	19,3

Variáveis contínuas foram analisadas pelo teste *t* de Student ou de Mann-Whitney, conforme o mais indicado. Variáveis qualitativas analisadas pelo teste do χ^2^ou exato de Fischer, conforme o mais apropriado. GC: Grupo Controle; GB: Grupo Butantan. Valores em percentis.

**Figura 2:** Valores da pressão média de vias aéreas, antes e após o tratamento, em ambos grupos de estudo

Em relação às complicações observadas durante a administração do surfactante, observou-se que menos crianças do Grupo Butantan apresentaram bradicardia em relação ao Grupo Controle (8,3%*versu* s 36,7%; p=0,002) tendo ambos os grupos apresentado similar incidência de refluxo de surfactante pela cânula traqueal (83,3%*versus* 76,7%; p=0,470).

## DISCUSSÃO

Este estudo multicêntrico teve como objetivo principal verificar a eficácia e a segurança do novo surfactante produzido pelo Instituto Butantan em relação aos produtos comercialmente disponíveis no Brasil. A mortalidade encontrada tanto com 72 horas de vida como no 28º dia de vida foi similar à observada com o uso dos dois surfactantes de origem animal utilizados no nosso meio. Essas observações demonstram que o principal benefício do uso de surfactante exógeno para o tratamento de recém-nascidos com SDR, ou seja, a redução da mortalidade, foi atingido. Esse achado tem sua importância bem estabelecida quando lembramos que, em várias maternidades públicas de nosso país, o surfactante exógeno não está disponível para uso clínico de rotina, em decorrência de seu elevado custo. De igual importância é o achado, em ambos grupos de estudo, de uma similar taxa de ocorrência das principais doenças relacionadas à prematuridade, incluindo a displasia broncopulmonar, a hemorragia intracraniana graus III e IV, e a enterocolite necrosante. Embora o Grupo Butantan tenha apresentado um menor boletim de Apgar de 5 minutos (7,9±1,5*versus*7,4±1,7; p=0,006), a magnitude da diferença não teve significado clínico.

Embora a incidência de pneumotórax tenha sido muito parecida nos dois grupos de estudo, as crianças tratadas com surfactante Butantan apresentaram uma maior incidência de enfisema intersticial pulmonar. Ambos os grupos apresentam uma incidência geral de ocorrência de ar extrapulmonar bem maior que a observada, tanto com o uso do lucinactante (um surfactante sintético aguardando aprovação do*Food and Drug Administration*− FDA) com o do poractante alfa (9,2% e 7,3%, respectivamente).^(13)^A resposta imediata à terapêutica com surfactante foi mais evidente no Grupo Controle, sendo avaliada por uma redução mais acentuada da pressão média de vias aéreas após o tratamento e com melhores valores de IO. No entanto, quando se analisa a variação desses parâmetros nos dois grupos estudados, constata-se que não foram associados a diferenças clínicas entre eles, fato demonstrado pela semelhança nos tempos de ventilação mecânica, tempo total de oxigênio e necessidade de oxigênio com 28 dias de vida, além da similar taxa de mortalidade com 72 horas e no 28º dia de vida, na incidência de hemorragia pulmonar e de pneumotórax.

A literatura internacional discute há cerca de duas décadas sobre a melhor forma de avaliar se um surfactante funciona adequadamente e como a eficácia entre dois surfactantes poderia ser comparada no tratamento de uma determinada doença respiratória. Com base em evidências publicadas, admite-se que os efeitos agudos do surfactante sobre os pulmões (avaliação da oxigenação) não predizem sua eficácia com clareza e são menos importantes do que a avaliação de sua influência na mortalidade associada à insuficiência respiratória.^(14-17)^A primeira grande evidência desse fato está nos estudos que compararam os surfactantes de origem animal aos sintéticos, em que fica claro que o surfactante utilizado para o tratamento melhora de função após sua exposição ao pulmão prematuro e sua modificação dentro do pneumócito II, com posterior liberação para o alvéolo.^(14)^Assim, a avaliação da mortalidade associada à insuficiência respiratória apresenta-se como o parâmetro mais importante na avaliação da eficácia da terapêutica com surfactante, quando comparada com a avaliação da oxigenação propriamente dita. A segunda grande evidência é mostrada com o uso de surfactante exógeno no tratamento da síndrome de aspiração meconial em recém-nascidos.^(18,19)^Nessa doença, a literatura internacional mostra que o tratamento com surfactante exógeno melhora a oxigenação a curto prazo. No entanto, apesar dessa melhora na oxigenação, estudos clínicos não confirmaram a redução da mortalidade na síndrome de aspiração meconial com a terapêutica com surfactante exógeno, fato este que torna o uso do surfactante, nessa doença, objeto de controvérsia e sem consenso.^(20)^Isso reforça a superioridade da avaliação da mortalidade na análise da eficácia da terapêutica com surfactante, em relação aos seus efeitos sobre a oxigenação.

Além da análise da eficácia, também se faz necessário avaliar a segurança desse novo medicamento a curto (no momento da administração) e a longo prazo (eventos que podem ocorrer como resultado de seu uso). Neste estudo, analisou-se a segurança de ambos os grupos no momento da sua administração (bradicardia e refluxo de surfactante pela cânula orotraqueal) e quanto à ocorrência de possíveis efeitos adversos associados aos mesmos. Os resultados obtidos mostraram que, durante a administração do surfactante, houve uma maior incidência de refluxo do surfactante pela cânula orotraqueal, entre os recém-nascidos do Grupo Butantan, e de bradicardia após a administração do surfactante, entre os recém-nascidos do Grupo Controle. Com relação aos efeitos adversos associados ao uso do surfactante, destaca-se a ocorrência de hemorragia pulmonar descrita na literatura internacional como o evento mais grave associado a esse tratamento.^(21)^Os resultados obtidos neste estudo mostraram que a hemorragia pulmonar ocorreu de forma semelhante nos dois grupos analisados.

Em relação à dose de surfactante utilizada neste estudo, um certo número de estudos clínicos e farmacocinéticos indica que doses maiores são mais eficazes.^(22)^A opção do uso da dose de 100 mg/kg foi baseada no fato de que, na SDR, inibidores da proteína derivada de soro e mediadores inflamatórios que inativam o sistema de surfactante se acumulam progressivamente, e o tratamento realizado na sua fase inicial é efetivo com a dose de 100mg/kg.^(23)^

Vários estudos comparando surfactantes sintéticos (sem proteínas em sua composição) e naturais (de origem tanto bovina como porcina) foram realizados, com resultados melhores entre os de origem animal em relação aos sintéticos. De modo semelhante aos resultados do presente estudo, as comparações entre diferentes surfactantes de origem animal (com as apoproteínas B e C em sua composição) não apresentaram resultados diferentes em relação à mortalidade e às principais morbidades relacionadas à prematuridade, incluindo parâmetros de ventilação mecânica, incidência de pneumotórax, enfisema intersticial, hemorragia intracraniana e persistência de canal arterial, quando comparados na mesma dose de tratamento.^(24,25,26)^

## CONCLUSÃO

O novo surfactante desenvolvido para o tratamento da síndrome do desconforto respiratório em recém-nascidos é eficaz e seguro, o que foi demonstrado por uma similar mortalidade neonatal e por uma comparável ocorrência de hemorragia pulmonar e de pneumotórax em ambos grupos de estudo.
